# Breaking the Myths Around e-Cigarettes: A Narrative Review Exploring the Impact of e-Cigarette on Human Health

**DOI:** 10.7759/cureus.72039

**Published:** 2024-10-21

**Authors:** Zareen Zohara, Ajeeth Rehman Abdul Jaffar Azad

**Affiliations:** 1 Internal Medicine, California Institute of Behavioral Neurosciences and Psychology, Fairfield, USA; 2 General Medicine, West Suffolk NHS Foundation Trust, Bury St Edmunds, GBR

**Keywords:** e-cigarettes, e-cigarette use in adolescents, human health, smoking, smoking cessation

## Abstract

In 2006, electronic cigarettes, or e-cigarettes, made their debut on the international scene. The term Electronic Nicotine Delivery Systems (ENDS) was introduced by the World Health Organization (WHO) in 2009 to refer to the many kinds of electronic cigarettes that include nicotine. This review article will provide a full investigation of the relationship between vaping and its effects on the body. The keywords utilized for search include "smoking cessation," "smoking," "e-cigarette use in adolescents," "human health," and "e-cigarettes." Our review goes into detail on the mechanism and components that make up a vaporizer. It explains how the components cause various side effects on human health. It is illustrated that vaping has various side effects and suggested that the risk of using vape should be educated to all. e-Cigarettes should be subjected to the same marketing limitations and establish quality standards for ingredients and functioning of e-cigarette devices. Thus, by deepening our understanding of these relationships and providing the results of the analysis accompanied by concise and meaningful illustrations to aid in understanding vaping and the physiological effects of the potential harm from vaping, we pave the way for future research on the quantity of puff to determine the harm level.

## Introduction and background

In 2006, electronic cigarettes (e-cigarettes), made their debut on the international scene [1]. The term Electronic Nicotine Delivery Systems (ENDS) was introduced by the World Health Organization (WHO) in 2009 to refer to the many kinds of electronic cigarettes that include nicotine [2]. Adults between the ages of 18 and 24 were found to be highest users of e-cigarette, and over half of them had never smoked cigarettes [3]. In recent years, vaping has become more common, especially among young adults (18-24 years old) and adolescents (11-17 years old). The introduction of new disposable devices has been largely blamed for this rise in vaping among these age groups [4]. Since they initially hit the market more than 10 years ago, ENDS have gained widespread acceptance as a less dangerous option to traditional cigarette smoking. Similar to smoking when referring to the usage of combustible tobacco cigarettes, the term "vaping" describes the practice of using e-cigarettes. Vaping has significantly risen worldwide due to the quick spread of innovative ENDS products and clever marketing strategies, raising substantial public health issues. More than 400 e-cigarette brands and more than 7000 flavour options were offered both in-store and online [5]. Since flavoured vaping liquid (or juice) is popular among teenagers, flavoured vape goods have been specifically targeted towards teenage consumers [6]. Because components like marijuana and concentrated tetrahydrocannabinol (THC) can be utilized in all devices that are now on the market, the use of these substances by adolescents has significantly increased. One in three high school kids and one in four middle school students who used e-cigarettes reported utilising cannabis in their devices, according to a 2018 California research [7].

The epidemic of teenage vaping use and the risk of nicotine addiction, the known and unknown risks to public health, and the unsolved questions surrounding the effectiveness of e-cigarettes as a harm reduction tool for tobacco users are among the urgent concerns that are addressed by a large portion of current research. Considering the known harm that tobacco use poses to global health, the questions of the safety and efficacy of e-cigarette use for smoking cessation require careful appraisal. There is a continuing dilemma about the safety of using e-cigarettes for smoking cessation and the effect they cause on the body.

Therefore, this review article will seek to provide a detailed analysis of the interplay between vaping and its effect on the body, with a focus on the mechanisms that link the two. In this way, by providing a detailed description of vaping and the physiological effects of the potential harm from vaping, we aim to improve our knowledge of this important relationship and, thus, create a basis for developing effective prevention interventions for reducing the misconceptions and the use of e-cigarettes in the modern world.

## Review

The mechanism behind e-cigarette function

E-cigarettes are electronic devices that operate via rechargeable batteries in the rechargeable ones and consist of an atomiser or heating element to heat the e-liquid and produce a vapour and consist of a cartridge filled with e-liquid [8]. They feature similar components but differ in size and shape. The various components in the e-cigarette and the products produced after heating are elaborated in Table 1 [9].

**Table 1:** The various components in the e-cigarette and the products produced after heating are elaborated e-liquid: electronic liquid e-cigarette: electronic cigarette

e-liquid contents	Device contents	After heating e-liquid (aerosol) contents	New components produced
Propylene glycol	Wire	Propylene glycol	Propylene oxide
Glycerol	Atomiser	Glycerol	Acrolein
Nicotine	Fibreglass wicks	Nicotine	Acetaldehyde
Flavouring		Flavouring	Formaldehyde
			Acetamide, copper, nickel, silver, silicate particles

Typically, the e-liquid comprises flavourings and humectants, either with or without nicotine. When the atomiser heats the liquid, the aerosol (vapour) creates a sensation like smoking tobacco, but it is said to have no negative consequences [10]. However, it is said that the process of heating can result in the creation of new, potentially dangerous chemicals [11].

Evolution of e-cigarettes

**Figure 1:**
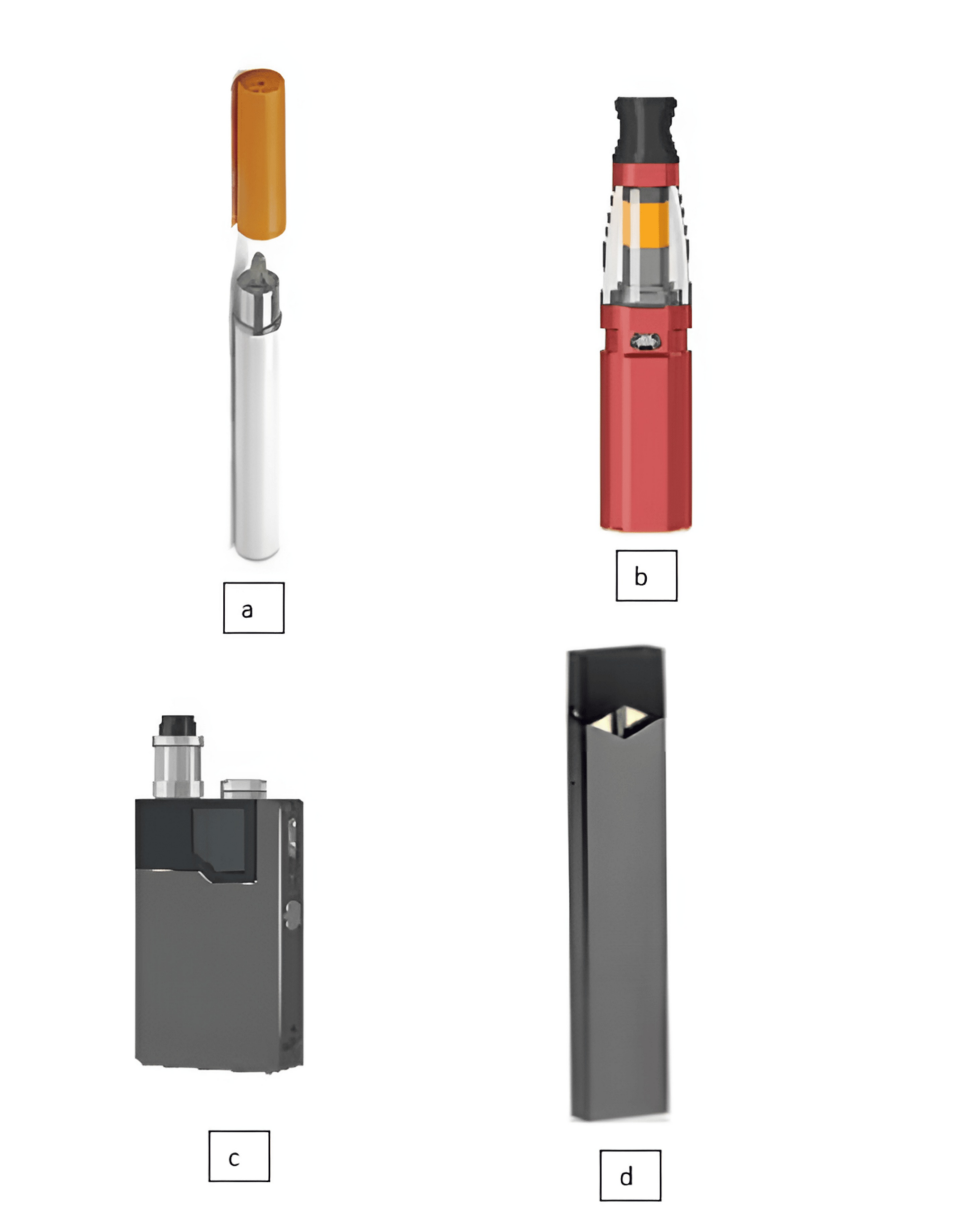
The different generations of e-cigarettes are shown a: Generation 1 b: Generation 2 c: Generation 3 d: Generation 4 Four generations of e-cigarette. Pulmonary effects of e-liquid flavors: a systematic review [11]. Journal of Toxicology and Environmental Health. This is an Open Access article distributed under the terms of the Creative Commons Attribution License Available: https://www.tandfonline.com/doi/figure/10.1080/10937404.2022.2124563?scroll=top&needAccess=true e-cigarettes: electronic cigarettes

Since the chemist Hon Lik began manufacturing e-cigarettes in China in 2003, the product's design has undergone rapid modifications. e-Cigarettes come in four different generations that vary in size, shape, and price. When e-cigarettes were first released, they looked a lot like regular cigarettes. The second generation, known as cleoatomizers, had a larger detachable tank that could hold e-liquid, a multi-voltage battery, and a detachable filament. The third-generation devices, often known as mods, are identified by modified batteries with variable voltages, wattages, and power capacities. The fourth-generation devices, such as USB flash drives, employ fixed-voltage batteries, which are shown in Figure 1 in a variety of shapes and sizes [12].

The e-cigarette device

A small number of studies have examined the material of the electronic device and its possible effects, but the majority of research on the effects of e-cigarette usage on human health has concentrated on the e-liquid components and the ensuing aerosols formed after heating. In this review, we have elaborated the effect of various components of the e-liquid and also the machine.

Nicotine

Being the most popular component and also a substance that's so deeply ingrained in our culture, it is very harmful to our health. Initially, 3-36 mg/mL of nicotine, a highly addictive substance found in tobacco leaves, was added to e-liquids [13]. Ionizing nicotine, on the other hand, made it more soluble and allowed for larger dissolved nicotine concentrations in e-liquids [14]. Nicotine salts are a kind of nicotine that is produced when freebase nicotine is lowered in pH by the addition of an acid (lactic, tartaric, salicylic, or benzoic) [15]. It has been reported that the resultant aerosol changes the pharmacokinetic profile of nicotine and is less unpleasant. Products using nicotine salts, which can contain more than 50 mg/mL of nicotine, are becoming more and more popular because of the decrease in unpleasant aerosol sensations and the rise in nicotine bioavailability [14].

Flavourings

Assessing the toxicity of e-cigarettes is complicated by the variety of compounds used to flavour e-liquids. There are various studies that focused on the possible effects of the flavouring which are shown in Table 2 [16,17].

**Table 2:** Various studies which focused on the possible side effects of flavouring e-liquids: electronic liquids e-cigarettes: electronic cigarettes

Author	Finding	The number of e-cigarettes analysed	Quantity of flavours present in e-cigarettes	Flavours present in e-cigarettes	Effect of flavorings
Hutzler et al. [16]	140 unique flavoring compounds	28 e-liquids	2.3 to 43 mg/mL	Vanillin, ethyl vanillin, ethyl maltol, and menthol	Cytotoxicity, irritation and inflammation, oxidative stress, respiratory diseases
Omaiye et al. [17]	150 chemicals	277 refill fluids	1 mg/mL	Ethyl maltol, menthol	Both taste additives were quite cytotoxic, carcinogenicity

Numerous studies have been conducted in vitro on the effects of popular flavourings, flavoured e-liquids, and flavourings with specific effects on cells [18]. All of this research on flavouring emphasises the necessity of laws limiting the amount of flavouring that may be found in e-cigarettes and outlawing flavours that are known to be harmful.

Humectants

Most e-liquids, such as propylene glycol and glycerol, serve as flavours and nicotine transporters in the creation of the vaping aerosol. Studies have revealed that propylene glycol can cause acute toxicity [19], cutaneous [20] and respiratory irritation, as well as change physiological systems [21].

Metals

The main possible source of exposure to harmful metals in e-cigarettes is the heating element. Metals such as manganese, cobalt, molybdenum, titanium, nickel, selenium, aluminium, and chromium have been found, along with other trace elements [22]. In general, if the wick is kept saturated with e-liquid, e-cigarette coil temperatures stay below 300°C. Coils can reach temperatures beyond 1000°C as the wicking material dries out, which accelerates coil breakdown and makes it easier for metals to be released [23].

New Components Produced

Acetone, propylene oxide, allyl alcohol, and acetaldehyde are the products of thermal degradation. At temperatures as low as 157°C, propylene glycol is oxidised to produce acetone, acetaldehyde, and formaldehyde. Studies have revealed the presence of aldehydes in the emissions from all e-cigarette generations, including formaldehyde, acetaldehyde, and acrolein which are carcinogens.

Impact of e-cigarette on health

e-Cigarettes are claimed to be safer than traditional cigarettes, according to a statement made by prominent health organisations, but the drawback is a lack of concrete proof of most items' negative effects. The health effects of e-cigarettes on various levels of the body are discussed in detail in the following sections. 

Oral Hazard

Aerosol-containing metals like formaldehyde and acrolein are carcinogenic. Oral tissues undergo biochemical alterations as a result of exposure to this. According to Menicagli et al., cancer has been identified as the primary disease route by emerging evidence of procarcinogenic changes linked to e-cigarettes [24]. This evidence includes DNA damage, RNA sequencing, and functional pathway analyses carried out on oral cells from vapers [25]. Additionally, following a 20-puff vaping session, a pilot study discovered that the expression levels of tumour suppressor and DNA repair genes were changed in the buccal cells of e-cigarette users [26]. Moreover, oral mucosal ulcers, periodontal disease, and dental caries are linked to e-cigarette use.

Nasal Hazards

The nasal epithelium has shown a significant drop in immune-related gene expression. This data suggests a decreased immune response, which raises the likelihood of bacterial or viral infections in e-cigarette users [27].

Effect on Lungs

Adults and adolescents who vape have a positive correlation with asthma, according to epidemiological research [28]. Repeated exposure to acrolein results in lung tissue damage caused by protease, reduced host defence, inflammation of neutrophils, mucus hypersecretion, and chronic pulmonary inflammation [29]. These effects are linked to the development of chronic obstructive pulmonary disease. The chance of developing chronic bronchitis symptoms is doubled in US high school juniors and seniors who use e-cigarettes [30].

Cardiovascular Effects

Nicotine enters the lungs through the ultrafine particles that are created by the aerosol. These particles are the same size as those seen in regular cigarettes. These minuscule particles exhibit biological activity, incite inflammatory responses, and have a direct correlation with the development of acute cardiovascular events and cardiovascular disease [31]. Even at modest levels of exposure to ultrafine particles, there is a significant increase in cardiovascular risk due to the nonlinear dose-response effect of particle exposure. Additionally, platelet adhesion, aggregation, and activation are induced by e-cigarette aerosol. A higher risk of cardiovascular disease is linked to all of these alterations. Short-term e-cigarette use also increases levels of biomarkers of oxidative stress. Long-term e-cigarette use is also associated with increased arterial stiffness and sympathetic tone. Cardiac-related effects from e-cigarette use include multiple acute hemodynamic changes, including impaired endothelial function, and increased blood pressure, heart rate, and sympathetic tone [32].

Neurological Effect

It also leads to nicotine addiction, which is a substance linked to chronic multidrug usage. Like with traditional cigarettes, the rapid brain absorption of nicotine from e-cigarettes can lead to addiction [33]. Numerous studies have demonstrated the "gateway effect" of early nicotine use, and high nicotine levels in many pod-based products, such as Juul and disposable e-cigarettes, cause users to become addicted quickly [34].

Effect on the Reproductive System

The New York Langone group observed that smoking e-cigarettes had reproductive health effects, including a drop in sperm count. According to the Centers for Disease Control, high nicotine levels in e-cigarettes can harm embryonic brain development in pregnancy [35].

e-Cigarettes as a tool for smoking cessation

With their distinct chemical compositions, e-cigarettes are largely replacing tobacco cigarettes in the market. According to producers, using an e-cigarette does not increase the risk of lung diseases like lung cancer, chronic obstructive pulmonary disease, or cardiovascular issues that are commonly related to regular cigarette usage [10]. The use of e-cigarettes seems to be a successful approach to quitting smoking, and it is linked to a reduced likelihood of negative effects compared to traditional tobacco cigarettes [36]. This is because nicotine-free e-cigarettes are now available, these products are being used as aids for quitting smoking.

Though there are reduced effects from e-cigarettes when compared with the traditional ones, there is an easy availability of e-cigarettes, which makes even the non-smokers try it for recreational purposes. With the use of e-cigarettes, there is a high chance for users to try traditional cigarettes, which in the meantime may be a factor for smoking addiction.

e-Cigarette and passive smoking

Several studies involving the adult population have revealed that e-cigarettes have short- and long-term consequences on respiratory health, even when exposed passively or second-hand to second-hand aerosol [37]. Passive exposure to e-cigarette aerosols has recently been linked to an increased risk of acquiring respiratory symptoms like dyspnea and bronchitis [38]. Islam et al. in their article reported a significant association between passive smoke from second-hand exposure to e-cigarettes and the risk of developing respiratory symptoms such as bronchitic symptoms or shortness of breath [39].

## Conclusions

e-Cigarettes have become widely used due to their easy availability and marketing strategies. Though it has been claimed to be more safe than traditional cigarettes, they have certain drawbacks and side effects likely to affect various organs in the body. They can also cause addiction and pave the way for the use of traditional cigarettes. In addition to this, vaping also can cause passive effects on non-vapers. There is also a high chance of keeping it carelessly and accessible to toddlers and children. Hence, we suggest that the use of vaping should also be monitored like traditional vaping and additional precautions should be taken to prevent second-hand smoking. We suggest that the risk of using vape should be educated to all. e-Cigarettes should be subjected to the same marketing limitations as conventional cigarettes, including no television, radio, or outdoor advertising. Quality standards for ingredients and functioning of e-cigarette devices need to be established. It is also recommended to work on a safe level of vape puffs per day and also suggested to avoid flavours as they can be a possible avoidable component causing side effects.
